# 

**Published:** 1862-02

**Authors:** 


### CONTENTS.

ORIGINAL ESSAYS AND COMMUNICATIONS.

A Few Facts in American Dentistry, Chronologically arranged. By DR. J. RICHARDSON, 53
Dental Periostitis—Treatment. By J. TAFT ........ 70
Facial Neuralgia. By W. H ATKINSON ........ 74
A Valuable Suggestion to those using Rubber Work in their Practice. By E. COLLINS... 81

SELECTIONS.

Practice of Hardening and Tempering Steel........ 83
Perchloride of Iron as an Escharotic ........ 86
Polishing Plates........ 87
Iodine........ 90

EDITORIAL.

The method of Using Artificial Teeth........ 94
The Vulcanite Patent Case........ 97
Vulcanizing Oven........ 99
Abscess........ 100
New Dental Depot........ 100

